# A Modified Flexor Tendon Suture Technique Combining Kessler and Loop Lock Flexor Tendon Sutures

**DOI:** 10.6061/clinics/2021/e2358

**Published:** 2021-04-26

**Authors:** Wenfeng Yang, Jvtao Li, Yuewen Su, Wu Liang, Yuanfei Ren, Yvjin Dong, Yaohua Shang, Sheng Zhong, Lianchun Xu, Tiehui Zhang

**Affiliations:** Dalian Municipal Central Hospital Affiliated of Dalian Medical University, Shahekou district, Dalian, Liaoning, China

**Keywords:** Flexor Tendon, Hand Surgery, Kessler-Loop Lock Suture, Biomechanical Property

## Abstract

**OBJECTIVES:**

In the present study, a novel single knot tenorrhaphy was developed by combining the modified Kessler flexor tendon suture (MK) with the loop lock technique.

**METHODS:**

A total of 48 porcine flexor digitorum profundus tendons were collected and randomly divided into six groups. The tendons were transversely cut and then repaired using six different techniques, the MK method, double knot Kessler-loop lock flexor tendon suture (DK), and single knot Kessler-loop lock flexor tendon suture (SK), each in combination with the epitendinous suture (P), and the same three techniques without P. Furthermore, by performing the load-to-failure tests, the biomechanical properties and the time taken to complete a repair, for each tenorrhaphy, were assessed.

**RESULTS:**

Compared to the MK+P method, DK+P was more improved, thereby enhancing the ultimate tensile strength. The SK+P method, which required fewer knots than DK+P, was easier to perform. Moreover, the SK+P repair increased the force at a 2-mm gap formation, while requiring lesser knots than DK+P.

**CONCLUSION:**

As opposed to the traditional MK+P method, the SK+P method was improved and exhibited better biomechanical properties, which may facilitate early mobilization after the repair.

## INTRODUCTION

A range of factors, including surface suture, suture materials, and different suture methods, impact the strength of flexor tendon repair. Currently, the optimal surgical approach for achieving successful results remains controversial. Flexor tendon injury continues to pose a number of challenges for hand surgeons. First, the injury cannot heal unless the two ends of the tendon are meticulously connected. Second, postoperative mobilization is critical to reduce local edema, prevent epitendinous adhesions, restore tendon strength, and improve gliding; however, this increases the risk of suture rupture and repair site gapping (1,2). The ideal repair method should be easy to perform, exhibit sufficient strength for healing, show minimal interference with tendon vascularity, and involve less suture knots with a smooth junction of the tendon ends (3).

The optimal suturing technique has long been investigated. Different suture materials and repair methods have been developed and tested by performing well-designed biomechanical experiments (4-6). The Kessler suture has undergone a wide range of modifications, and has been the most commonly used repair approach (7). However, identifying the optimal surgical approach for achieving successful results remains challenging. Although various suture techniques have been used clinically (8-11), the modified Kessler flexor tendon suture (MK) has rarely been combined with the loop lock technique (12). Compared with grasping loops, locking loops were capable of improving strength and gap resistance in flexor tendon repair by drawing upon 2-0 and 3-0 core sutures. However, these advantages disappeared when 4-0 core suture material was used (13).

Several studies have reported that grasping loops (MK) combined with locking loops show the advantages of both the suture techniques and dramatically enhance the biomechanical properties of tendon repair (14,15). Chinese researchers developed the double knot Kessler-loop lock flexor tendon suture (DK) technique (16), which is capable of significantly enhancing the strength as compared with the modified Kessler methods. Moreover, double knot Kessler-loop lock flexor tendon suture combined with the epitendinous suture (DK+P) was proven to exhibit better biomechanical properties at different time points of tendon healing, with no significant interference with tendon vascularity. It is considered a reliable and effective alternative for repairing severe contusions of tendon tissues. This technique has two knots in the broken tendon contact surface, which could probably elevate the risk of knot loosening, suture slippage, and tendon torsion. Therefore, in the present study, the DK method was modified by altering the double knot to a single knot, and the biomechanical properties and the time taken to complete a repair were compared among the traditional modified Kessler sutures.

## METHODS

### Tendon harvest and repair

Since their structure and diameter are similar to those of human flexor tendons, porcine flexor tendons were used in this study for tendon suture research and biomechanical studies. In total, 48 porcine flexor digitorum profundus tendons (mean circumference: 8-10 mm) were collected from the hind legs (weighing approximately 300-400 g) of adult pigs (Dalian Chuming Co., Ltd.) and randomly divided into six groups (eight tendons in each group). All operations in this study were performed in compliance with the international guidelines for the care and treatment of experimental animals. A 4-0 nylon stitch (Weigao, China) was used as the core suture, and a 6-0 nylon stitch (Weigao, China) was selected for the epitendinous sutures.

**Figure 1:**
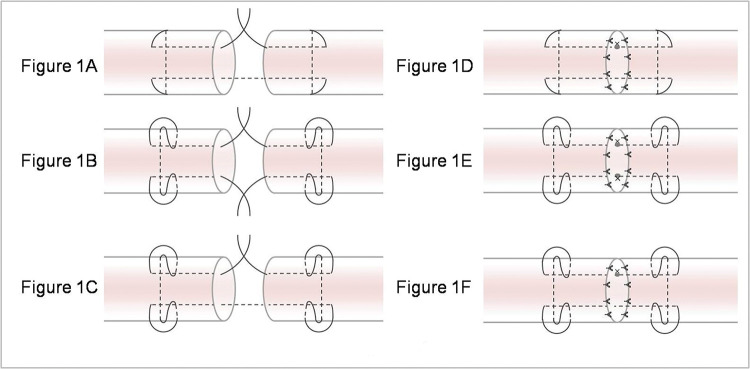
Various suture techniques used for mechanical testing. (A) Modified Kessler suture without epitendinous suture (MK); (B) double knot Kessler-loop lock flexor tendon suture without epitendinous suture (DK); (C) single knot Kessler-loop lock flexor tendon suture without epitendinous suture (SK); (D) Modified Kessler suture with epitendinous suture (MK+P); (E) double knot Kessler-loop lock flexor tendon suture with epitendinous suture (DK+P); (F) single knot Kessler-loop lock flexor tendon suture with epitendinous suture (SK+P).

The tendons were transversely cut in the middle with a scalpel and then repaired with six different techniques: (A) Modified Kessler suture without epitendinous suture (MK), (B) double knot Kessler-loop lock flexor tendon suture without epitendinous suture (DK), (C) single knot Kessler-loop lock flexor tendon suture without epitendinous suture (SK), (D) Modified Kessler suture with epitendinous suture (MK+P), (E) DK+P, and (F) single knot Kessler-loop lock flexor tendon suture with epitendinous suture (SK+P). Figure 1 illustrates these techniques.

To provide proper tension, the sutures in the first locking loop were tightened during the Kessler suture, and the final range of the stitches was maintained on both sides at 10-12 mm. The surgical knots were employed for all suture knots, comprising six alternating positive and negative single knots (Figure 1). To ensure test accuracy and comparability, all repairs were performed by a single experienced surgeon in one day. The time required for suturing was recorded in each group. After suturing, the repaired tendons were covered with wet (normal saline) gauze and stored at 4°C overnight. Subsequently, they underwent biomechanical testing the following morning.

### Load-to-failure tests

**Figure 2:**
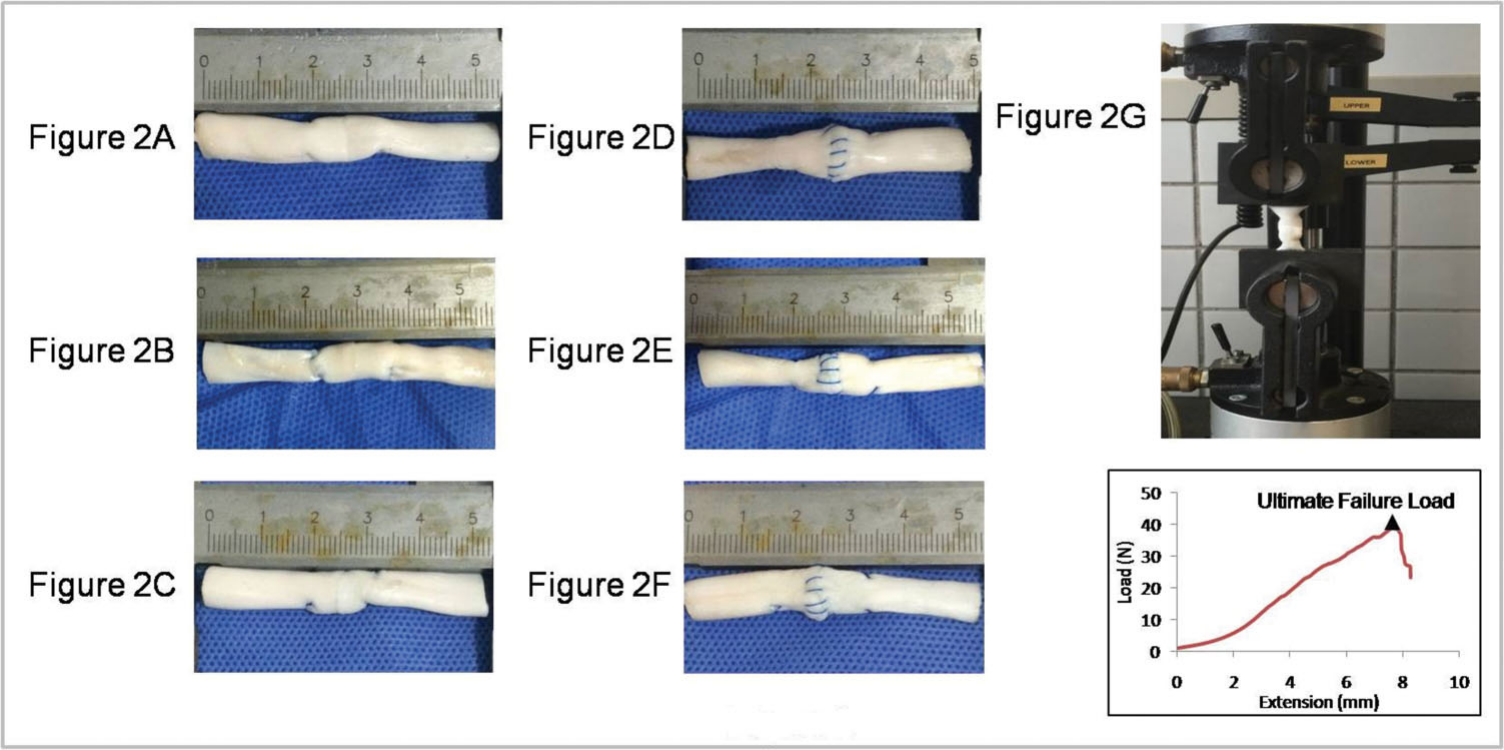
Repaired porcine tendon with various suture techniques. (A) Modified Kessler suture without epitendinous suture (MK); (B) double knot Kessler-loop lock flexor tendon suture without epitendinous suture (DK); (C) single knot Kessler-loop lock flexor tendon suture without epitendinous suture (SK); (D) Modified Kessler suture with epitendinous suture (MK+P); (E) double knot Kessler-loop lock flexor tendon suture with epitendinous suture (DK+P); (F) single knot Kessler-loop lock flexor tendon suture with epitendinous suture (SK+P). (G) The repaired porcine tendon in mechanical testing and the Load-deformation curve in test.

The tendon samples were cut down to 50 mm in length, with the repair site in the middle, and the ends of the tendon were clamped at a distance of 15 mm from the repair site in a testing machine (5567AModel, Instron, UK) (Figure 2). The Bluehill software (Instron, UK) was used to set and record the test parameters to ensure that each tendon underwent a preload of 1.0 N and was then elongated at a constant velocity of 10 mm/min, until the suture site was completely ruptured. The load-to-failure tests were captured using a camera. During the test, the gap at the repair site was measured with a Vernier caliper, and the 2-mm Gap Load was defined as the tensile force required to form a 2-mm gap at the repair site. Furthermore, the load-displacement curve was plotted using a computer, and the ultimate load was recorded as the peak force during the stretch.

### Statistical analysis

SPSS18.0 (IBM Corporation, US) was employed for all the statistical analyses. In addition, analysis of variance (ANOVA) was adopted to assess the differences in the 2-mm Gap Load, Ultimate Failure Load, and suture times between the six groups; subsequently, the post hoc Tukey’s test was performed. Data are expressed as mean±standard deviation (SD), and *p*<0.05 indicated statistical significance.

## RESULTS

### 2-mm Gap Load

**Figure 3:**
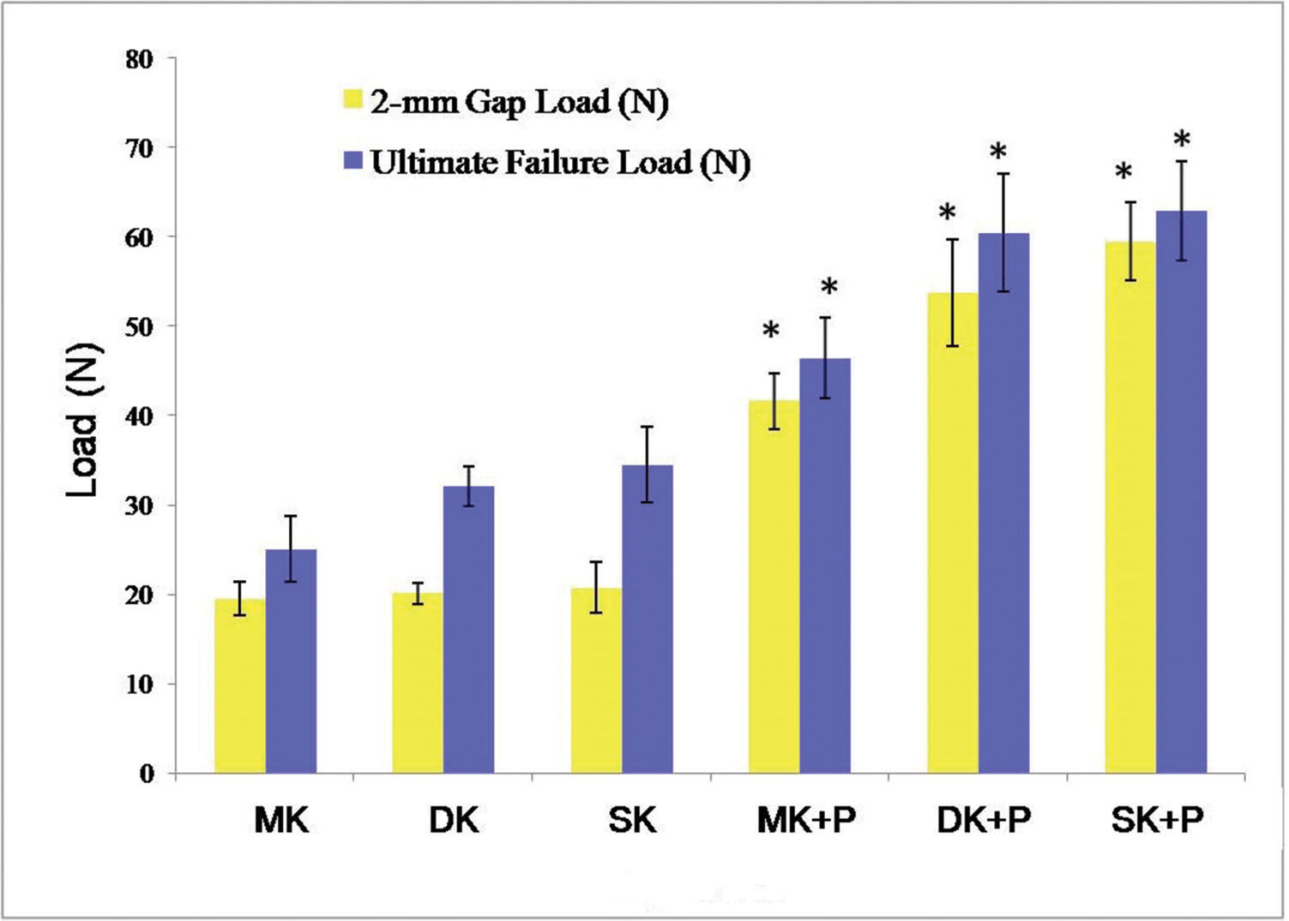
The 2-mm Gap Load and Ultimate Failure Load of the six suture techniques. In comparison with single core suture method, the combination of core and epitendinous sutures withstood a significantly increased 2-mm Gap Load and Ultimate Failure Load. Error bars represented the SD from eight porcine flexor tendons. *p*<0.05 *versus* core suture, respectively. MK, modified Kessler suture without epitendinous suture; DK, double knot Kessler-loop lock flexor tendon suture without epitendinous suture; SK, single knot Kessler-loop lock flexor tendon suture without epitendinous suture; MK+P, modified Kessler suture with epitendinous suture; DK+P, double knot Kessler-loop lock flexor tendon suture with epitendinous suture; SK+P, single knot Kessler-loop lock flexor tendon suture with epitendinous suture; SD, standard deviation.

The 2-mm Gap Load reached 19.5±1.9 N, 20.1±1.2 N, 20.8±2.9 N, 41.6±3.2 N, 53.7±6.0 N, and 59.5±4.4 N in MK, DK, SK, MK+P, DK+P, and SK+P, respectively (Table 1, 2 and Figure 3). As opposed to the single core suture methods, the combination of core and epitendinous sutures significantly increased the 2-mm Gap Load (*p*<0.001). No significant difference was identified in the 2-mm Gap Load among the three core suture methods (*p*>0.05). The 2-mm Gap Load was higher in DK+P and SK+P than in MK+P (*p*<0.001). Moreover, SK+P achieved a greater 2-mm Gap Load than DK+P (*p=*0.03).

### Ultimate Failure Load

Table 1, 3 and Figure 3 present that the Ultimate Failure Load reached 25.0±3.7 N, 32.1±2.2 N, 34.5±4.3 N, 46.5±4.5 N, 60.4±6.6 N, and 62.9±5.6 N in MK, DK, SK, MK+P, DK+P, and SK+P, respectively. The core sutures combined with the epitendinous sutures withstood a higher Ultimate Failure Load than single core sutures (*p*<0.001). Likewise, DK (*p=*0.046) and SK (*p=*0.003) withstood a higher Ultimate Failure Load than the MK method. Furthermore, DK+P and SK+P withstood a higher Ultimate Failure Load than MK+P (*p*<0.001). Nevertheless, the Ultimate Failure Load was nearly consistent between DK and SK (*p=*0.901) or between DK+P and SK+P (*p=*0.897).

### Failure Profile

**Figure 4:**
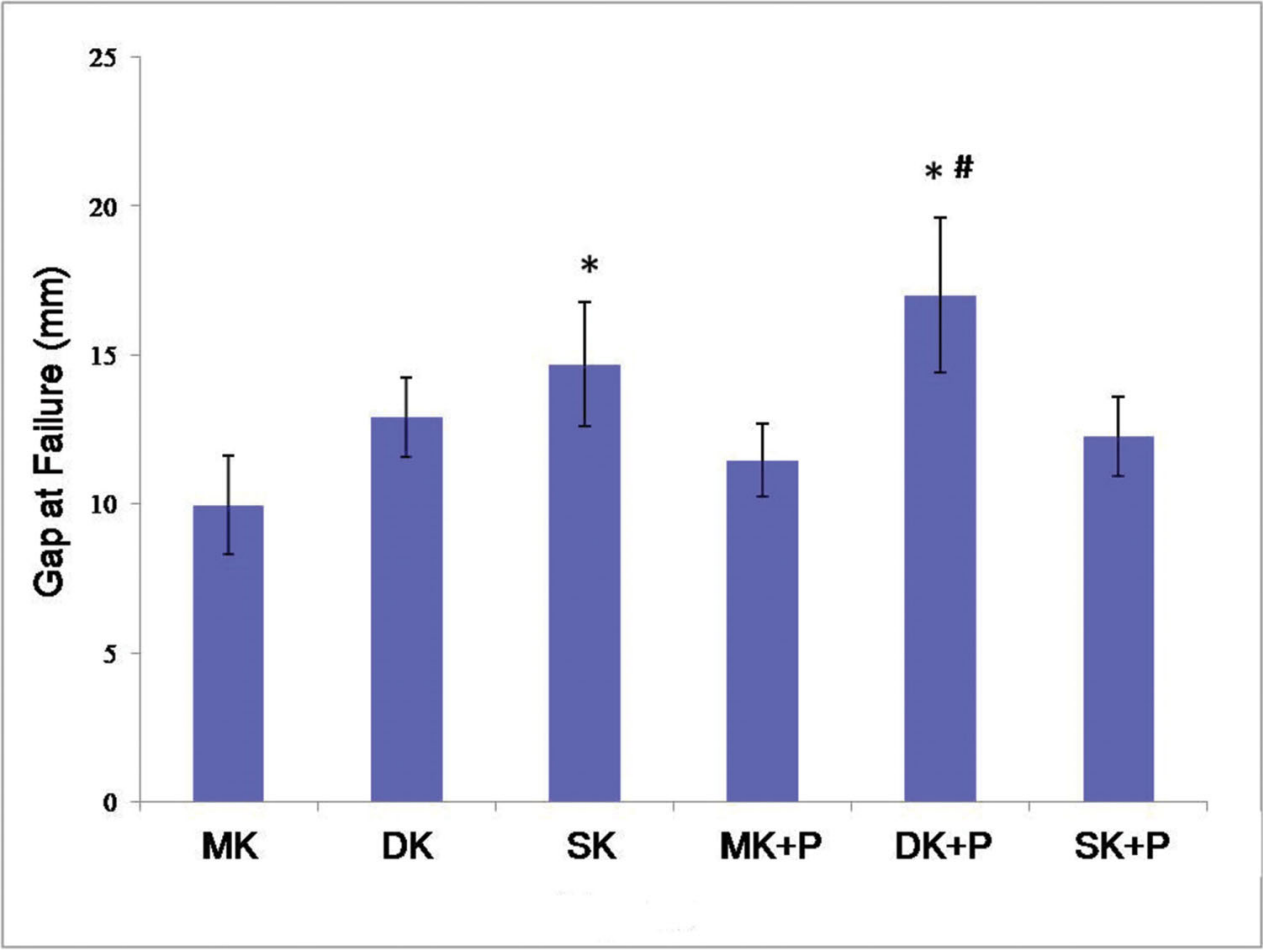
The gap at failure of the six suture techniques. The lowest gap at failure was observed in the MK group (10.0 mm), while the largest was in the DK+P group. There were no statically significant differences between MK and DK, MK+P, and SK+P (*p*>0.05). However, SK and DK+P improved the gap at failure significantly. As compared to DK combined with epitendinous suture, there was a smaller gap at failure in the MK+P and SK+P groups. *p<0.05 *versus* MK # *p*<0.05 *versus* MK+P group. MK, modified Kessler suture without epitendinous suture; DK, double knot Kessler-loop lock flexor tendon suture without epitendinous suture; SK, single knot Kessler-loop lock flexor tendon suture without epitendinous suture; MK+P, modified Kessler suture with epitendinous suture; DK+P, double knot Kessler-loop lock flexor tendon suture with epitendinous suture; SK+P, single knot Kessler-loop lock flexor tendon suture with epitendinous suture.

The suture mostly ruptured at the root of the knot in the MK, DK, and SK groups, while a small portion broke at the gap site of the tendon. In the MK+P, DK+P, and SK+P groups, the epitendinous running suture broke at first always in the gap site, some at the root of the knot. With an increase in the tensile strength, the core sutures began to break. The gap at failure was showed in Figure 4.

### Time Required

**Figure 5:**
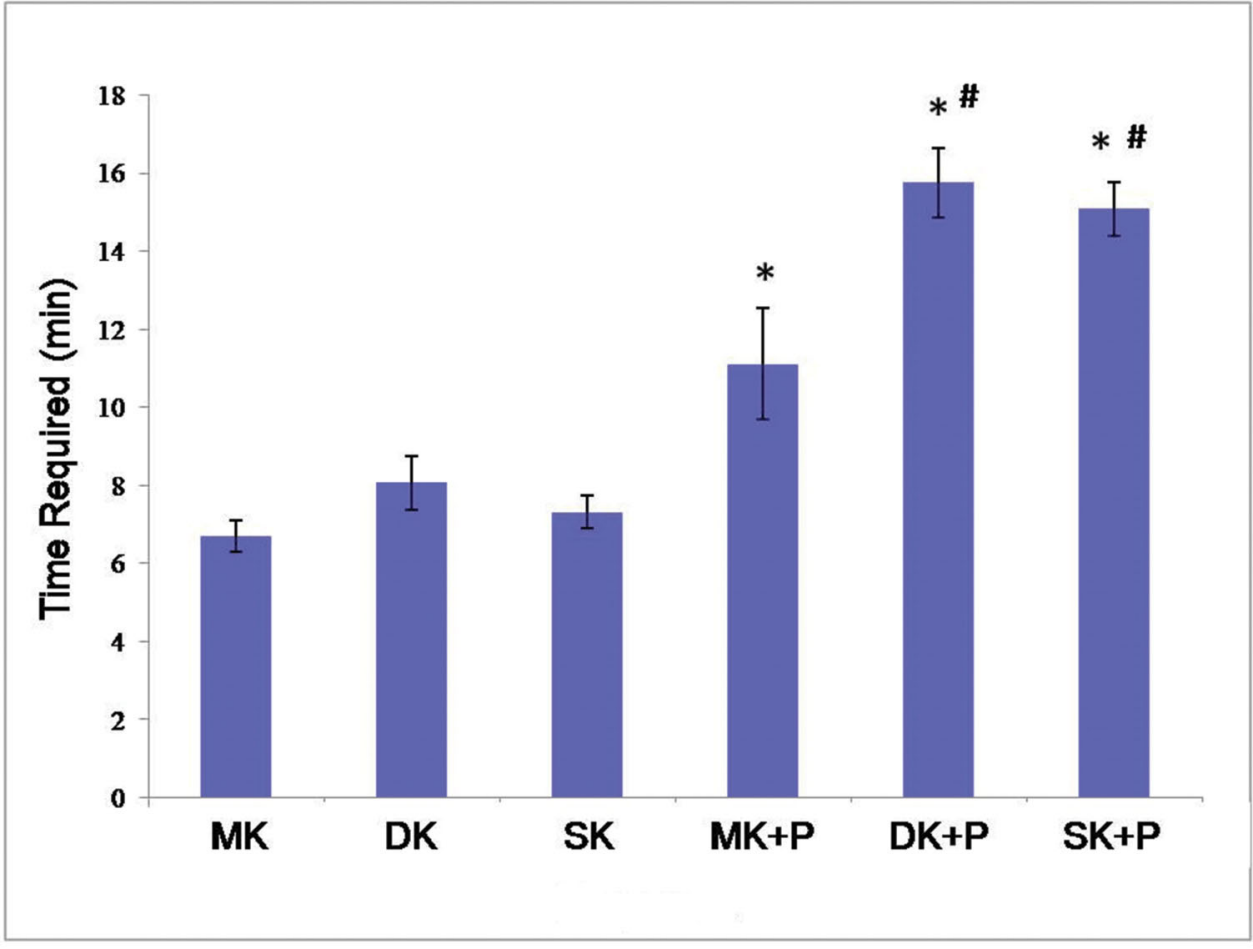
The time required for the six suture techniques. It took a longer time to perform MK+P, DK+P, and SK+P than to perform single core sutures (*p*<0.05). And the suture time was significantly longer for DK+P and SK+P than MK+P (*p*<0.05). However, there was no significant difference between DK+P and SK+P (*p*>0.05). *p<0.05 *versus* core suture # *p*<0.05 *versus* MK+P. MK, modified Kessler suture without epitendinous suture; DK, double knot Kessler-loop lock flexor tendon suture without epitendinous suture; SK, single knot Kessler-loop lock flexor tendon suture without epitendinous suture; MK+P, modified Kessler suture with epitendinous suture; DK+P, double knot Kessler-loop lock flexor tendon suture with epitendinous suture; SK+P, single knot Kessler-loop lock flexor tendon suture with epitendinous suture.

According to Table 1 and Figure 5, the time required for MK, DK, SK, MK+P, DK+P, and SK+P reached 6.7±0.4 min, 8.0±0.7 min, 7.3±0.4 min, 11.1±1.4 min, 15.8±0.9 min, and 14.6±0.5 min, respectively. MK+P, DK+P, and SK+P took a longer time to be performed than the single core sutures (*p*<0.05). Moreover, the suture time was significantly longer for DK+P and SK+P than for MK+P (*p*<0.05). However, no significant difference was identified between DK+P and SK+P (*p*>0.05).

## DISCUSSION

It has been proven that peripheral epitendinous sutures can increase repair strength and reduce gapping between tendon ends. The core suture exhibited the first resistance to gap formation, while the epitendinous sutures grasped both the epitenon and the tendon substance to form a smooth tendon surface and decreased the repair bulk (17,18). As revealed from the results of the biomechanical testing in the present study, the core sutures combined with the epitendinous sutures could withstand a higher 2-mm Gap Load and Ultimate Failure Load than the single core sutures, demonstrating that an epitendinous suture could critically increase the 2-mm Gap Load.

According to the results, both DK+P and SK+P could significantly withstand a stronger 2-mm Gap Load and Ultimate Failure Load (over 50 N) than MK+P. Epitendinous sutures have been reported to improve gap resistance and the force of repair by 10-50% (19-21). During tendon healing, a gap formation of >2 mm was associated with adhesion formation, decreased tendon gliding, and poor clinical results (22). Therefore, the 2-mm Gap Load should be higher than or equal to the actual force (approximately 1-29 N by active flexion without resistance) of inactive finger movement during early rehabilitation (23). In the present study, MK+P, DK+P, and SK+P provided over 30 N of strength. The mentioned strengths were similar to those of other approaches, such as 10-strand and double Kessler (24). Although the 10-strand and double Kessler techniques could provide sufficient strength, the complex configuration and time-consuming procedure probably hinder tendon vascularity and healing (25,26).

Biomechanical testing suggested the superiority of the Kessler-loop lock flexor tendon suture over the traditional MK method. In addition, as opposed to DK+P, SK+P could endure a higher 2-mm Gap Load; however, no significant difference was identified in the ultimate tensile strength between the two methods. It took over 14 min to perform DK+P and SK+P, which was significantly longer than the time required for MK+P. Nevertheless, no remarkable difference was found in the time required for suturing between DK+P and SK+P. Therefore, SK+P is simpler to perform and can exhibit better biomechanical properties for healing than DK+P.

The following tips should be considered for the performance of SK+P (1). The suture should be run longitudinally in the palmar 1/3 of the tendon during core suture, in an attempt to reduce the interference with tendon vascularity and facilitate endogenous tendon healing (2). During core suture, after the completion of the suture on one side and before the suture of the other side, the stitch should be tightened to close the tendon ends and exert a certain pre-load (3). Additionally, an epitendinous suture should be performed to enhance the mechanical properties of tendon sutures. The ends of the tendon fibers should be buried in the epitendinous sutures to reduce tendon adhesions and enhance endogenous healing (4). The cross-sectional area of the tendon lock should be 1/8-1/6 of the total fiber section on each side. A larger area is likely to reduce the blood supply, whereas an extremely limited tendon lock area leads to insufficient tensile strength.

There are some limitations to the present study. First, the use of porcine tendons may not be a perfect approximation of actual clinical results. In addition, all sutured testing was performed with a two-strand core suture, not resembling a typical modern-day repaired clinical suture that commonly utilizes four or more strands. It is very useful to assess the biomechanical properties of loop lock techniques with human tendons and other suture materials. In conclusion, compared with MK+P, SK+P was stronger, and it enhanced the ultimate tensile strength and effectively prevented gap formation. SK+P repair could increase the force at a 2-mm gap formation, while requiring fewer knots than DK+P, which may be conducive to early mobilization after the repair.

## AUTHOR CONTRIBUTIONS

YangWperformed biomechanical testing, analyzed the data, and wrote the manuscript. Li J and Su Y harvested the porcine flexor digitorum profundus tendons. Liang W, Ren Y, and Dong Y contributed to the editing of the manuscript. Shang Y, Zhong S, and Xu L contributed to the collection of experimental data. Zhang T directed this study. All authors read and approved the final manuscript.

## Figures and Tables

**Table 1:** The 2-mm Gap Loads and Ultimate Failure Loads of the six suture techniques.

Group	2-mm Gap Load (N)		Ultimate Failure Load (N)		Time Required (min)	
	Mean	SD	Mean	SD	Mean	SD
MK	19.5	1.9	25.0	3.7	6.7	0.4
DK	20.1	1.2	32.1	2.2	8.0	0.7
SK	20.8	2.9	34.5	4.3	7.3	0.4
MK+P	41.6	3.2	46.5	4.5	11.1	1.4
DK+P	53.7	6.0	60.4	6.6	15.8	0.9
SK+P	59.5	4.4	62.9	5.6	14.6	0.5

MK, modified Kessler suture without epitendinous suture; DK, double knot Kessler-loop lock flexor tendon suture without epitendinous suture; SK, single knot Kessler-loop lock flexor tendon suture without epitendinous suture; MK+P, modified Kessler suture with epitendinous suture; DK+P, double knot Kessler-loop lock flexor tendon suture with epitendinous suture; SK+P, single knot Kessler-loop lock flexor tendon suture with epitendinous suture; SD, standard deviation.

**Table 2:** *p*-values for 2-mm Gap Loads of the six suture techniques.

	MK	DK	SK	MK+P	DK+P	SK+P
MK		1.000	0.988	<0.001	<0.001	<0.001
DK	1.000		1.000	<0.001	<0.001	<0.001
SK	0.988	1.000		<0.001	<0.001	<0.001
MK+P	<0.001	<0.001	<0.001		<0.001	<0.001
DK+P	<0.001	<0.001	<0.001	<0.001		0.030
SK+P	<0.001	<0.001	<0.001	<0.001	0.030	

MK, modified Kessler suture without epitendinous suture; DK, double knot Kessler-loop lock flexor tendon suture without epitendinous suture; SK, single knot Kessler-loop lock flexor tendon suture without epitendinous suture; MK+P, modified Kessler suture with epitendinous suture; DK+P, double knot Kessler-loop lock flexor tendon suture with epitendinous suture; SK+P, single knot Kessler-loop lock flexor tendon suture with epitendinous suture.

**Table 3:** *p*-values for Ultimate Failure Loads of the six suture techniques.

	MK	DK	SK	MK+P	DK+P	SK+P
MK		0.046	0.003	<0.001	<0.001	<0.001
DK	0.046		0.901	<0.001	<0.001	<0.001
SK	0.003	0.901		<0.001	<0.001	<0.001
MK+P	<0.001	<0.001	<0.001		<0.001	<0.001
DK+P	<0.001	<0.001	<0.001	<0.001		0.897
SK+P	<0.001	<0.001	<0.001	<0.001	0.897	

MK, modified Kessler suture without epitendinous suture; DK, double knot Kessler-loop lock flexor tendon suture without epitendinous suture; SK, single knot Kessler-loop lock flexor tendon suture without epitendinous suture; MK+P, modified Kessler suture with epitendinous suture; DK+P, double knot Kessler-loop lock flexor tendon suture with epitendinous suture; SK+P, single knot Kessler-loop lock flexor tendon suture with epitendinous suture.
